# ﻿Two new host records for *Centrodoraitalica* Ferrière (Hymenoptera, Aphelinidae) from eggs of Tettigoniidae (Orthoptera, Ensifera) in northeastern Italy

**DOI:** 10.3897/zookeys.1156.97364

**Published:** 2023-03-24

**Authors:** Giacomo Ortis, Serguei V. Triapitsyn, Luca Mazzon

**Affiliations:** 1 Department of Agronomy, Food, Natural Resources, Animals and Environment (DAFNAE), University of Padova, Legnaro, Italy University of Padua Padua Italy; 2 Entomology Research Museum, Department of Entomology, University of California, Riverside, California, USA University of California Riverside United States of America

**Keywords:** Aphelinid, egg parasitoid, Mediterranean region, parasitic wasp, sentinel eggs, tettigoniid

## Abstract

The egg parasitoid *Centrodoraitalica* Ferrière is reported for the first time from sentinel eggs of two species of Tettigoniidae (Orthoptera), *Pachytrachisgracilis* (Brunner von Wattenwyl) and *Eupholidopteraschmidti* (Fieber). In Italy, only two hosts of this parasitic wasp are known, one of which is a tettigoniid species. Exposure of sentinel eggs represented a useful method to detect new host associations of this parasitoid species that can search for their host’s eggs in the ground. The parasitoids were identified by comparing our specimens with those of the type series, and the original description of *C.italica*.

## ﻿Introduction

The genus *Centrodora* Förster, 1878 (Hymenoptera, Aphelinidae) is cosmopolitan and includes parasitoids which develop on insects belonging to different orders (Hayat 1974; Polaszek 1991; Viggiani 1994; Bocca et al. 2020). In particular, some members of *Centrodora* are known to parasitize eggs of Orthoptera and Hemiptera, whereas other species can attack nymphs of Hemiptera, and pupae of Diptera, Hymenoptera and Coleoptera. The checklist of the Italian fauna includes four species of *Centrodora*: *C.brevifuniculata* Viggiani, 1972, *C.cicadae* Silvestri, 1918, *C.italica* Ferrière, 1968 and *C.livens* (Walker, 1851) (www.faunaitalia.it). Three of these species are recorded from hemipteran hosts at egg or pupal stage, while only *C.italica* was reared from eggs or pupae of species belonging to various orders including *Uromenusbrevicollis* (Fischer, 1853) (Orthoptera, Tettigoniidae), *Saperdacarcharias* (L., 1758) (Coleoptera, Cerambycidae), *Euderuscaudatus* Thomson, 1878 (Hymenoptera, Eulophidae) (Cavalcaselle 1968; Noyes 2019). Recently, *C.livens* was reared in Italy from eggs of the introduced Nearctic pest *Metcalfapruinosa* Say, 1830 (Hemiptera, Flatidae) (Raspi and Canovai 2003).

Here we report new host records for *C.italica* from sentinel eggs of two tettigoniid species.

## ﻿Materials and methods

During summer 2021, five species of Tettigoniidae were collected in multiple localities in the Euganean and Berici Hills of the Veneto Region of Italy: *Pachytrachisgracilis* (Brunner von Watternwyl, 1861), *Pholidopteralittoralis* (Fieber, 1853), *Pholidopterafallax* (Fischer, 1853), *Eupholidopteraschmidti* (Fieber, 1861) and *Decticusalbifrons* (F., 1775). About thirty total individuals of both sexes were reared in cages in a greenhouse with temperature gradually fluctuating between 19 °C and 35 °C in relation to natural photoperiod (15 h light) and relative humidity cycling between 70% and 80%. Each cage contained, at the bottom, a tray filled with washed sand for egg laying. Adults were fed with branches of *Rubus* sp., various fruits and vegetables, and also with dry cat food. On 6 August 2021, eggs of all five tettigoniid species (at least 13 for each species) were sifted from sand and placed in a permanent meadow in the Friuli Venezia Giulia Region, Italy (Udine Province, near Godia: 46°06'21.9"N, 13°16'38.5"E, 127 m a.s.l.) under bushes of *Rubus* sp., approximately 2 cm beneath the soil surface. To prevent damage by predators, sentinel eggs of each species were placed in one soil-filled plastic cup (10 × 10 × 5 cm) covered with a nylon net of 0.9 mm mesh. The bottom of the cup was removed and replaced with a nylon net to allow for rainwater flow. All the eggs were retrieved on 25 September 2021 and incubated in a laboratory at room temperature in plastic vials on moist filter paper. All collecting and rearing was done by G. Ortis.

The parasitoids were subsequently identified by S. V. Triapitsyn as *C.italica* by directly comparing them with the type series of this species and the original description, since it was made after the publication of the key to the European species of the genus by Ferrière (1965).

## ﻿Results

**Figure 1.**
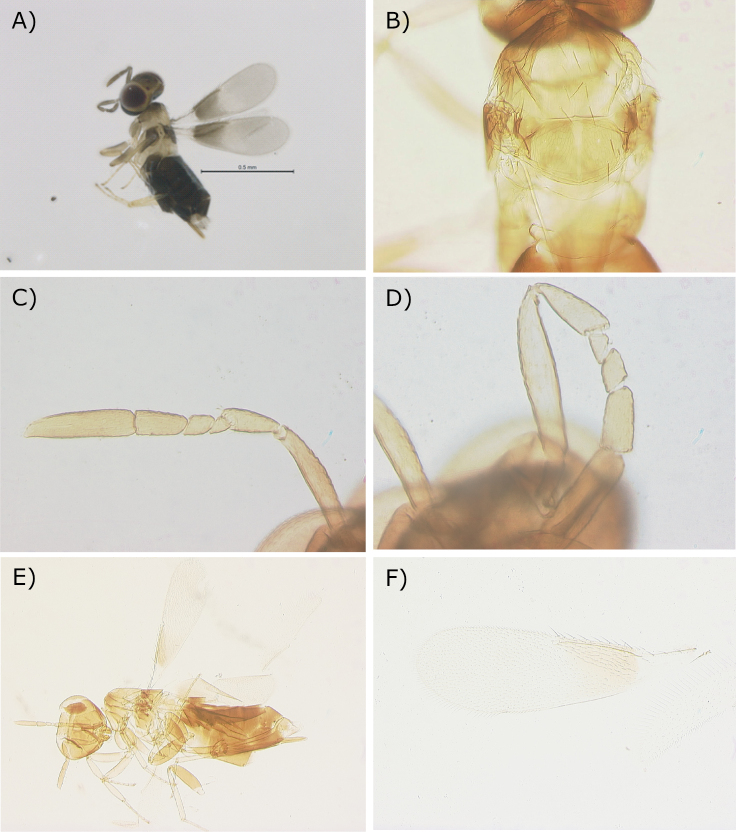
*Centrodoraitalica* from this study **A** habitus of female (in ethanol) **B** mesosoma **C** antenna **D** antenna **E** habitus (slide-mounted specimen) **F** fore wing. Scale bar: 0.5 mm.

On 22 November 2021, seven adult parasitoids hatched from each of two eggs of *Eupholidopteraschmidti* and seven adult parasitoids hatched from each of two eggs of *Pachytrachisgracilis*. Although females reared by us have a relatively shorter and less protruding ovipositor (Fig. 1) than those of the type series of *C.italica* from another tettigoniid host, we consider that likely to be subject of intraspecific variability rather than warranting description of a new species in the genus where recognition of species worldwide, including Europe, is already extremely difficult. Females of *C.italica* from our study are illustrated here (Fig. 1) to facilitate their recognition.

### ﻿Taxonomic remarks

**Figure 2.**
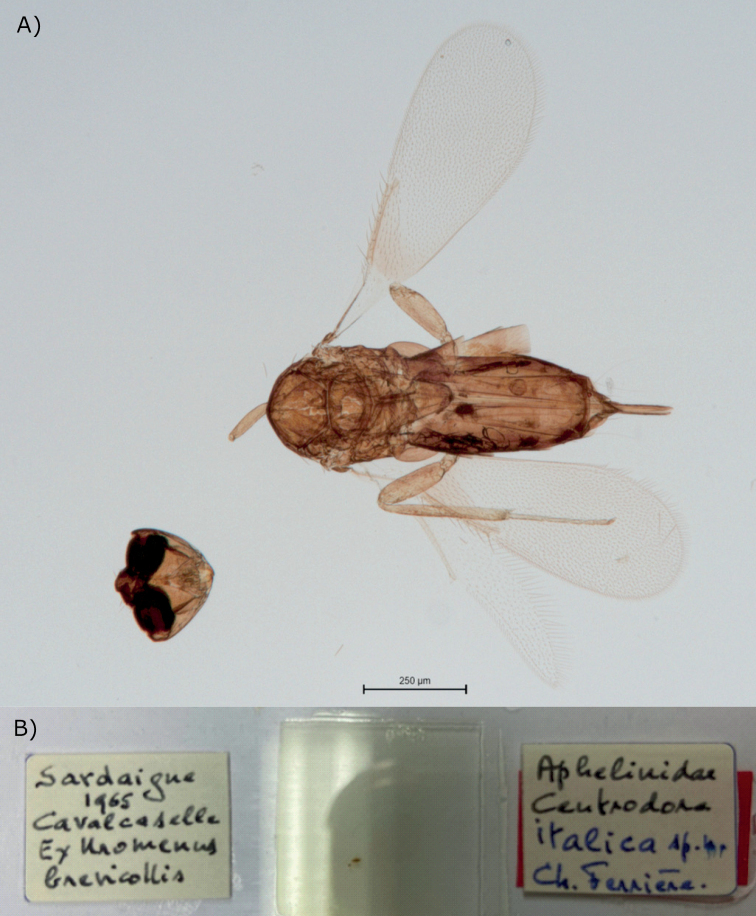
*Centrodoraitalica*, female of the type series **A** habitus **B** slide. Scale bar: 0.25 mm.

**Figure 3.**
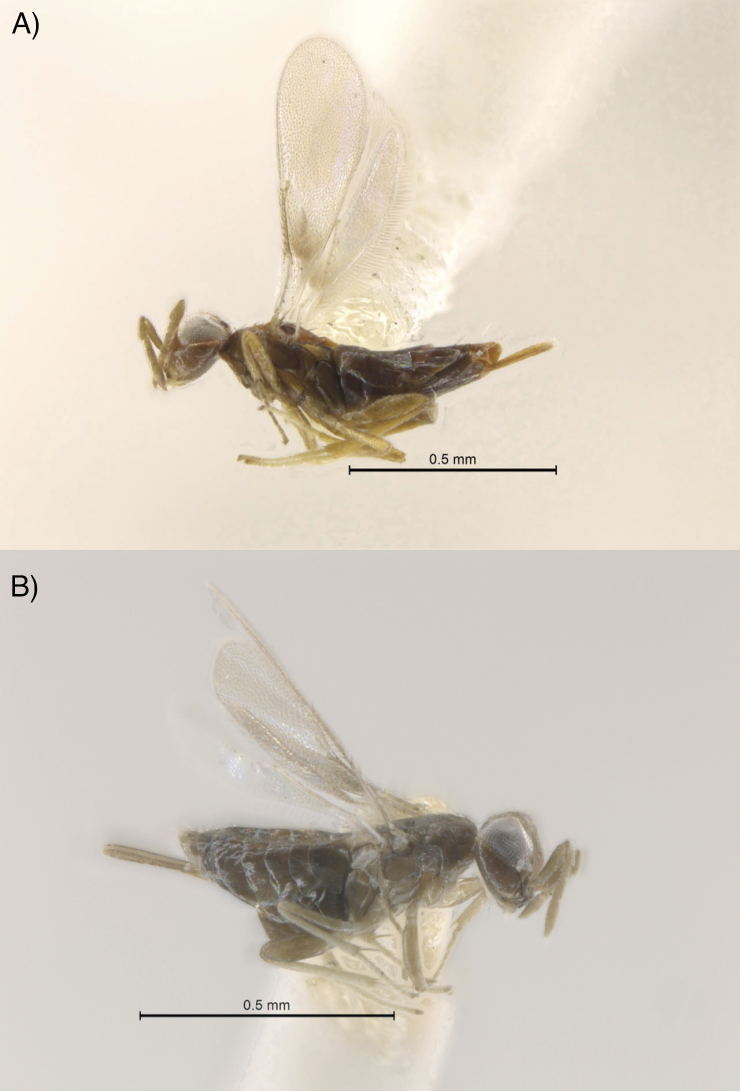
*Centrodoraamoena*: **A***C.amoena* female from Manosque, France **B***C.amoena* female from Ural area, Russia. Scale bar: 0.5 mm.

Ferrière (1968) described *C.italica* from the holotype female from Sardinia and several paratypes of both sexes from Sardinia and Rome, Italy. In the collection of Muséum d’Histoire Naturelle de la Ville de Genève, Geneva, Switzerland (**MHNG**), the type series of this species is mounted on five slides, four of which are labeled identically as follows: (1) “Sardaigne 1965 Cavalcaselle Ex *Uromenusbrevicollis*”, (2) “Aphelinidae*Centrodoraitalica* sp. n. Ch. Ferrière.”; the remaining slides are labeled as follows: (3) “Italie Rome 1965 Cavalcaselle Ex *Uromenusbrevicollis*”, and (4) “Aphelinidae*Centrodoraitalica* sp. n. Ch. Ferrière.”. Apparently, B. Cavalcaselle was the collector in Sardinia, but the exact type locality on the island is unknown. All specimens are in very poor condition, being dissected and slide-mounted without prior clearing and missing most of the antennae. Because the holotype was not marked, it is impossible now to decide which one of those specimens from Sardinia was the actual primary type. The best preserved and most complete slide-mounted female (1) is illustrated here (Fig. 2) for comparing purposes. Proper recognition of *C.italica* would require support from molecular evidence, as morphologically this species, particularly females from Sardinia and Rome, seem to be very similar to the widespread European species *C.amoena* Förster, 1878 (Fig. 3). The distinguishing features between these two nominal species mentioned by Ferrière (1968) in the diagnosis of *C.italica* are all of minor nature and eventually may be proven to be within the range of intraspecific variability of *C.amoena*.

### ﻿Other material examined

*Centrodoraamoena* (specimens in the National Museum of Natural History, Washington DC, USA [USNM]): France, Alpes-de-Haute-Provence, Manosque, 1959, H. L. Parker, “mass rearing alfalfa stems”, 1 female. Russia, with the following labels: “Ural, 1931”, “Egg pods of Locustids”, “Rec’d from M. N. Nikolskaya let[ter]., 5-17-1935”, 6 females.

*Centrodoraitalica* (specimen in the Entomology Research Museum, University of California, Riverside, California, USA [UCRC]): Italy, Campania, Caserta Province, SE end of Lago del Matese, 41°24.41'N, 14°24.20'E, 1050 m, 7.vi.2003, M. Bologna, J. Munro, A. Owen, J. D. Pinto, 1 male.

## ﻿Discussion

Here we report the first record of *Centrodoraitalica* from northeastern Italy and two new host records of this species. Both *Eupholidopteraschmidti* and *Pachytrachisgracilis* occur from northeastern Italy and southernmost Austria across parts of the Balkans to Bulgaria and Greece, and are usually found in the transition zone of shrubs and small clearings (IUCN 2021). The wide distributional area of these hosts suggests that this parasitoid could be present also in other areas in the Mediterranean region.

While the common collection methods for micro-hymenopterous parasitoids, such as Malaise traps, yellow pan traps, sweep netting or Berlese funnels, allow for the capture of these wasps at adult stage, they are not suitable to detect their host associations, in particular for those species that search for their hosts in the ground (Ortis et al. 2020). Because we placed sentinel eggs in August, we can hypothesize that the activity of this parasitoid takes place during summer after, or during, the oviposition period of most tettigoniid species (Ingrisch 1986; Ortis et al. 2022). It will be worth, for future studies, to provide genetic data for species delimitation within this genus *Centrodora*.
